# Acute Upper Limb Ischemia Due To Arterial Thrombosis in a Mild COVID-19 Patient: A Case Report

**DOI:** 10.7759/cureus.10349

**Published:** 2020-09-10

**Authors:** Muhammad Hanif, Mukarram Jamat Ali, Muhammad Adnan Haider, Sidra Naz, Zeeshan Ahmad

**Affiliations:** 1 Internal Medicine, Hayatabad Medical Complex, Khyber Medical College, Peshawar, PAK; 2 Internal Medicine, King Edward Medical University, Lahore, PAK; 3 Internal Medicine, Allama Iqbal Medical College, Lahore, PAK; 4 Internal Medicine, University of Health Sciences, Lahore, PAK; 5 Internal Medicine, Khyber Teaching Hospital, Peshawar, PAK

**Keywords:** covid-19, sars-cov-2, limb ischemia, arterial thrombosis, coagulopathy

## Abstract

Coronavirus disease 2019 (COVID-19) which has now been declared a global pandemic, initially began as a pneumonia caused by novel coronavirus called severe acute respiratory syndrome-coronavirus-2 (SARS-CoV-2) in Wuhan, China. COVID-19, in addition to respiratory symptoms, is also being recognized to have different manifestations including myocardial infarction, seizures, meningitis, diarrhea, and coagulopathy. Here we report a case of a 75-year-old female patient with mild COVID-19 who later developed acute limb ischemia due to arterial thrombosis to highlight that, contrary to the association of coagulopathy with severe COVID-19, thrombosis can also occur in patients with mild COVID-19

## Introduction

In December 2019, cases of pneumonia began to spread in Wuhan, China, and a novel coronavirus was isolated from these patients that was later named the severe acute respiratory syndrome-coronavirus-2 (SARS-CoV-2) in January 2020 [1]. The pneumonia disease caused by SARS-CoV-2 was named coronavirus disease 2019 (COVID-19) by the World Health Organization (WHO) and was declared a global pandemic on March 11, 2020 [2]. COVID-19 was initially considered to be a respiratory disease with symptoms ranging from mild fever, dry cough, and sore throat to acute respiratory distress syndrome [3], but now cardiovascular [4], neurological [5], and gastrointestinal [6] complications are being increasingly recognized as COVID-19 symptoms. The association of coagulopathy with COVID-19 has been demonstrated by elevated levels of D-dimer and prolonged prothrombin time in the patients with severe COVID-19 and by autopsies that show that 58% of cases have deep vein thrombosis, 30% of which also had fatal venous thromboembolism [7]. The incidence of arterial thrombosis, albeit very low in comparison to venous thrombosis, has also been reported in patients with COVID-19. Although the exact incidence of COVID-19 associated thrombotic events has yet to be determined, these events range from pulmonary embolism to limb ischemia [8,9]. The trend of hypercoagulability associated with severe COVID-19 needs to be investigated, as hypercoagulability is associated with poor prognosis of the disease. Here, contrary to reports in the literature that suggest coagulopathy is associated with severe disease, we present a case of an older woman with clinically mild COVID-19 who underwent thrombectomy of the radial and ulnar thrombus to prevent permanent limb ischemia.

## Case presentation

A 75-year-old woman presented to the emergency department (ED) with a history of bluish discoloration of the left hand along with pain and numbness lasting one day. On examination, she was alert, oriented, and vitally stable. Her left hand was cold with bluish discoloration of the fingers. She had a positive brachial pulse, an absent ulnar pulse, and a positive but feeble radial pulse. She had 60% oxygen saturation in her left middle finger and 0% in all other fingers of her left hand. Table 1 and Table 2 present her laboratory workup.

**Table 1:** Preoperative laboratory findings

Test	Result
Hemoglobin	11.4 g/dL
Total lymphocyte count	5.9 (x10^9^/L)
Red blood cell count	4.5 (x10^12^/L)
Platelets	733 (x10^9^/L)
Prothrombin time	13 seconds
Activated partial thromboplastin time	25 seconds
D-dimer	867 ng/FEUmL (reference value: up to 500 ng/FEUmL)
Blood urea	45 mg/dL
Creatinine	1 mg/dL
Sodium	142 mEq/L
Potassium	4.3 mEq/L

**Table 2:** Postoperative laboratory findings

Tests	Results
Hemoglobin	11.5 g/dL
Total lymphocyte count	8.5 (x10^9^/L)
Red blood cell count	4.5 (x10^12^/L)
Platelets	580(x10^9^/L)
Prothrombin time	12.4 seconds
Activated partial thromboplastin time	28.3 seconds
D-dimer	643 ng/FEUmL
Blood urea	52 mg/dL
Creatinine	1 mg/dL
Sodium	141 mEq/L
Potassium	4.6 mEq/L

Due to our suspicion of acute limb ischemia, Doppler ultrasound of the left arm was conducted, revealing an absent blood flow in radial and ulnar arteries consistent with thrombosis. She was started on intravenous antibiotics, heparin, and analgesics. Her past medical history was significant for COVID-19, diagnosed by polymerase chain reaction for SARS-CoV-2 via nasopharyngeal swab one week prior to presentation to the ED when she developed typical symptoms of low-grade fever and flu. However, at the time of her presentation, her symptoms had resolved. Her thrombophilic profile (i.e., antithrombin 3 level, factor 5 mutation, protein C deficiency, and protein S deficiency) and anti-neutrophilic antibodies (ANA) were assessed.

Echocardiogram showed no intracardiac thrombus and a normal ejection fraction of 52%. Meanwhile, the pain and numbness of her left hand worsened, and she had both left radial and ulnar thrombectomy under strict hygienic control. She was stable postoperatively and was discharged on the third postoperative day with instructions to follow-up in one week. Her thrombophilic screen and ANA were negative. Given the absence of a previous history of such incident, no recent history of left arm/hand trauma, and no history of atrial fibrillation, she was diagnosed with a possible thrombotic complication secondary to SARS-CoV-2.

## Discussion

Cantador et al., in a retrospective observational study, demonstrated that out of 1419 COVID-19 patients, 14 (1%) patients developed systemic arterial thrombotic events of acute coronary syndrome, acute ischemic stroke, and acute lower limb ischemia with a mortality rate of 28.6% in these patients [10]. Similarly, a recent observational cohort study from Italy illustrated an increase in the incidence of acute limb ischemia in patients with COVID-19 from January to March 2020 compared to January to March 2019 (16.3% vs. 1.8%, respectively; p<.001) [11]. Likewise, in another observational study, 3.7% of the critically ill intensive care unit patients with COVID-19 were found to have arterial thrombotic events [8].

Several mechanisms have been proposed to delineate the pathophysiology of coagulopathy associated with COVID-19 [12]. First, viral invasion of vascular endothelium and endotheliitis may play a role. One key mechanism that results in vasoconstriction and end-organ ischemia is the direct invasion of endothelial cells by the virus using angiotensin-converting enzyme 2 (ACE2) receptors on the endothelial cells and subsequent endotheliitis leading to endothelial dysfunction. Viral inclusions have also been observed in the endothelial cells of the patients with COVID-19 [13].

A hypercoagulable state may arise from the action of inflammatory cytokines. Severe COVID-19 has been associated with a cytokine profile characterized by increased production of interleukin-1, interleukin-2, interleukin-6, and tumor necrosis factor-alpha [5]. These inflammatory cytokines activate platelets, endothelium, neutrophils, and result in thrombosis [12].

Another mechanism in the pathophysiology of coagulopathy associated with COVID-19 involves monocytes, macrophages, and neutrophils. Activated monocytes in severe COVID-19 upregulate the tissue factor, which in turn activates the coagulation cascade and thrombosis [12]. Neutrophil extracellular trap generation from abnormally activated neutrophils turns on the cytokine storm and thrombosis [12]. Lastly, the immobilization of critically ill patients results in stasis and hypercoagulability.

Contrary to the literature, which shows that thrombosis mostly occurs in severe COVID-19 patients, our patient did not have severe COVID-19; instead, she had a mild disease characterized by a low-grade fever and flu-like symptoms that resolved the day before presentation to the ED. On presentation, she had no COVID-19 related symptoms; her only presenting signs were bluish discoloration of the left hand along with pain and numbness. In the absence of common etiologies associated with the development of arterial blockage (by either thrombosis or embolus) such as peripheral arterial disease (atherosclerotic disease), vascular trauma, atrial fibrillation, acute myocardial infarction [14], the subtle direct endothelial damage and activation caused by SARS-CoV-2 appears to be the only explanation for our patient’s condition. In this patient, the inflammatory cytokines and activated monocytes and neutrophils did not appear to have a role in the activation of the coagulation cascade leading to arterial thrombosis because the patient was stable both clinically and on laboratory findings. Thus, this case underscores that direct endothelial invasion, damage, and activation caused by SARS-CoV-2 using ACE2 receptors with subsequent coagulation activation is the vital underlying pathophysiological mechanism that leads to the development of thrombosis.

## Conclusions

This case emphasizes the importance of monitoring the patients of advanced age with COVID-19 for the development of arterial thrombotic complications even if they do not have severe COVID-19. We also recommend the stratification of patients with COVID-19 based on their risk of development of arterial thrombosis and the use of prophylactic antithrombotic treatment accordingly.

## References

[1] Lu H, Stratton CW, Tang YW (2020). Outbreak of pneumonia of unknown etiology in Wuhan, China: the mystery and the miracle. J Med Virol.

[2] Wang C, Horby PW, Hayden FG, Gao GF (2020). A novel coronavirus outbreak of global health concern. Lancet.

[3] Chen N, Zhou M, Dong X (2020). Epidemiological and clinical characteristics of 99 cases of 2019 novel coronavirus pneumonia in Wuhan, China: a descriptive study. Lancet.

[4] Kang Y, Chen T, Mui D (2020). Cardiovascular manifestations and treatment considerations in COVID-19. Heart.

[5] Ahmed MU, Hanif M, Ali MJ (2020). Neurological manifestations of COVID-19 (SARS-CoV- 2): a review. Front Neurol.

[6] Gu J, Han B, Wang J (2020). COVID- 19: gastrointestinal manifestations and potential fecal-oral transmission. Gastroenterology.

[7] Zamboni P (2020). COVID-19 as a vascular disease: lesson learned from imaging and blood biomarkers. Diagnostics.

[8] Klok FA, Kruip MJHA, van der Meer NJM (2020). Incidence of thrombotic complications in critically ill ICU patients with COVID-19. Thromb Res.

[9] Fu L, Wang B, Yuan T (2020). Clinical characteristics of coronavirus disease 2019 (COVID-19) in China: a systematic review and meta-analysis. J Infect.

[10] Cantador E, Núñez A, Sobrino P (2020). Incidence and consequences of systemic arterial thrombotic events in COVID-19 patients. J Thromb Thrombolysis.

[11] Bellosta R, Luzzani L, Natalini G (2020). Acute limb ischemia in patients with COVID-19 pneumonia. J Vasc Surg.

[12] Abou-Ismail MY, Diamond A, Kapoor S, Arafah Y, Nayak L (2020). The hypercoagulable state in COVID- 19: Incidence, pathophysiology, and management. Thromb Res.

[13] Varga Z, Flammer AJ, Steiger P (2020). Endothelial cell infection and endotheliitis in COVID-19. Lancet.

[14] Engledow AH, Crinnion JN (2002). Acute lower limb ischaemia. Hosp Med.

